# Regulatory Mechanisms between Quorum Sensing and Virulence in *Salmonella*

**DOI:** 10.3390/microorganisms10112211

**Published:** 2022-11-09

**Authors:** Xiaojie Zhang, Baobao Liu, Xueyan Ding, Peng Bin, Yang Yang, Guoqiang Zhu

**Affiliations:** 1 Institute of Comparative Medicine, College of Veterinary Medicine, Yangzhou University, Yangzhou 225009, China; 2 Joint Laboratory of International Cooperation on Prevention and Control Technology of Important Animal Diseases and Zoonoses of Jiangsu Higher Education Institutions, Yangzhou University, Yangzhou 225009, China; 3 Jiangsu Co-Innovation Center for Prevention and Control of Important Animal Infectious Diseases and Zoonoses, Yangzhou University, Yangzhou 225009, China; 4 Joint International Research Laboratory of Agriculture and Agri-Product Safety, The Ministry of Education of China, Yangzhou University, Yangzhou 225009, China

**Keywords:** *Salmonella*, virulence, quorum sensing, signaling molecules, quorum quenching

## Abstract

*Salmonella* is a foodborne pathogen that causes enterogastritis among humans, livestock and poultry, and it not only causes huge economic losses for the feed industry but also endangers public health around the world. However, the prevention and treatment of *Salmonella* infection has remained poorly developed because of its antibiotic resistance. Bacterial quorum sensing (QS) system is an intercellular cell–cell communication mechanism involving multiple cellular processes, especially bacterial virulence, such as biofilm formation, motility, adherence, and invasion. Therefore, blocking the QS system may be a new strategy for *Salmonella* infection independent of antibiotic treatment. Here, we have reviewed the central role of the QS system in virulence regulation of *Salmonella* and summarized the most recent advances about quorum quenching (QQ) in virulence attenuation during *Salmonella* infection. Unraveling the complex relationship between QS and bacterial virulence may provide new insight into the therapy of pathogen infection.

## 1. Introduction

*Salmonella* is a foodborne pathogen that causes bacterial gastroenteritis in both humans and livestock or poultry [1]. The data show that there are 93 million *Salmonella* infectious cases around the world each year, and more than 155,000 people die from *Salmonella* infection each year, which brings great challenges to public health and the food industry [2]. The pathogenicity of *Salmonella* is closely related to the its virulence factors, including biofilm formation (BF), flagellar-mediated motility, adhesion and invasion, and the type III secretion system (T3SS) [3,4,5]. Antibiotic treatment is one of the common therapies for *Salmonella* infection [6,7]. Unfortunately, with increased frequency of antibiotic application, *Salmonella* has developed serious resistance to antibiotics. In this context, a new antibiotic-free antibacterial therapy is urgently required to treat *Salmonella* infection.

The quorum sensing (QS) system is an intercellular cell–cell communication mechanism that coordinates bacterial adaptation to the environment. Many bacteria can produce and release QS molecules into the environment, which allows bacteria to respond to their own and environmental conditions by receiving signal molecules from the environment and themselves [8]. With a threshold concentration of QS molecules reached, the receptor protein interacts with the QS molecules which induces a signaling pathway that consequently modulates cellular metabolic processes in response to environmental changes. QS affects the biosynthesis and assembly of pef fimbria by regulating the expression of *Salmonella* (the resistance to complement killing) *rck* locus [9]. The cellular processes are associated with bacterial virulence and survival of bacteria in their environment [10,11], such as biofilm formation, flagellar-mediated motility, adhesion and invasion, andT3SS [9,12]. Consequently, inhibition of *Salmonella* QS has become a new and promising antibacterial strategy, which not only resists *Salmonella* virulence but also prevents the development of bacterial resistance. In this article, we described the formation of different QS in *Salmonella*, discussed regulatory mechanisms between QS and *Salmonella* virulence, and focused on a new strategy for using the QS system to combat *Salmonella* infection. Such a strategy may have the potential to be a promising next-generation therapy to control *Salmonella* infection and tackle the development of antibiotic resistance.

## 2. Different Signal Molecules Mediate QS in *Salmonella*

**Figure 1:**
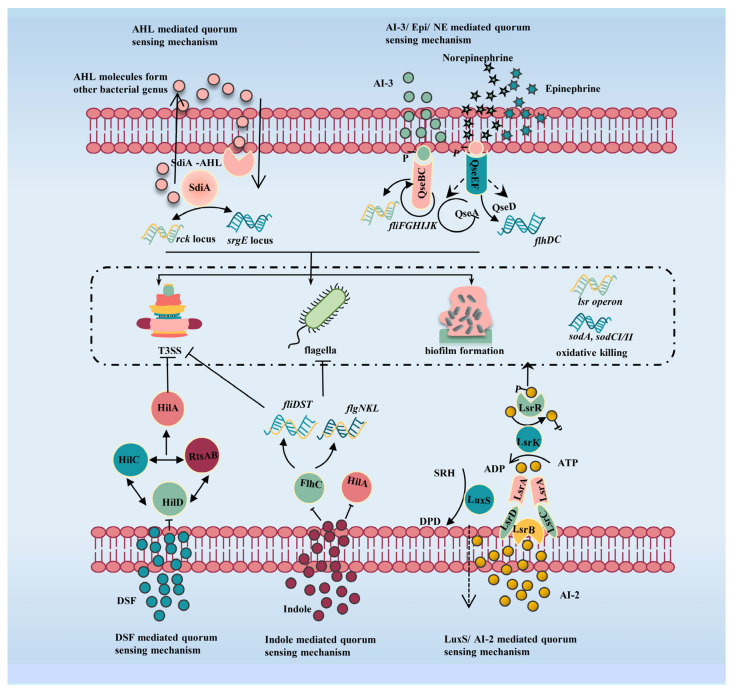
Regulatory mechanisms between QS and virulence in *Salmonella*. AHL-mediated QS regulated the expression of the *srgE* and *rck* locus through the SdiA receptor, which in turn affected the expression of the T3SS genes and bacterial invasion. LuxS/AI-2-mediated QS regulated the expression of flagella-related genes (*fliC*, *fliD*, and *flgL*), oxidative stress-response genes (*sodA*, *sodCI,* and *sodCII*), and TS33-related genes (*sipA*, *sipC*, and *sipD*), which in turn affected bacterial invasion. AI-3/Epi/NE-mediated QS regulated the expression of T3SS genes, which in turn affected bacterial invasion, flagella-related genes, and it can also regulate biofilm formation. DSF-mediated QS inhibited the expression of *hliD* thus inhibiting the expression of SPI-1. Indole-mediated QS inhibited the expression of *hliA* thus inhibiting the expression of SPI-1 and also inhibited the expression of *flhC* thus inhibiting flagella biosynthesis.

In *Salmonella*, there are five types of QS signal molecules that contribute to the regulation of bacterial survival and virulence. These include Autoinducer (AI)-1, AI-2, AI-3/Epinephrine (Epi)/Norepinephrine (NE), diffusible signal factors (DSF), and indole (Figure 1). Acyl Homoserine Lactones (AHLs), generally known as AI-1, are a common QS signal molecule in Gram-negative bacteria. AHLs are synthesized through *S*-adenosylmethionine (SAM) and acyl–acyl carrier protein in the presence of protein members of the LuxI family of AHL synthase. Interestingly, although *Salmonella* cannot synthesize AHLs by itself, it can still sense AHLs signal generated from other microorganisms by the SdiA (a LuxR homolog) of *Salmonella*, and control a wide variety of traits [3]. For example, AHL produced by *Aeromonas hydrophila* and *Yersinia enterocolitica* in the host intestine activates the AHL receptor SdiA which promotes intestinal colonization [13]. In addition, SdiA can directly and positively regulate *rck* binding to AHL which regulates bacterial invasion of host cells [14]. AI-2 is another type of main QS signal molecule in *Salmonella*, which is produced through the conversion of *S*-adenosyl homocysteine (SAH) by the AI-2 synthase LuxS [15]. The LuxS-dependent AI-2 activates the expression of *lsr* operon, which is composed of seven genes (*lsrACDBFGE*) and is responsible for AI-2 uptake in *Salmonella* [12,16]. Recent studies have shown that the transcriptional regulator LsrR can repress the expression of genes, such as *sodA*, *sodCI*, and *sodCII*, whose function is associated with *Salmonella* survival within macrophages, which contributes to the virulence of *Salmonella* [17]. Intriguingly, *Salmonella* can also utilize the QS signal molecules produced by other organisms, such as AI-3, Epi, and NE. AI-3 is a bacterial signal produced by the intestinal commensal microflora, while Epi and NE are the stress hormones of hosts [18,19]. The *Salmonella* express a histidine kinase (HK) sensor called QseC, which can recognize the AI-3/Epi/NE signal and further mediate bacterial virulence. It is reported that QseC activation upregulates the transcriptional expression of genes related to the colonization and motility in *Salmonella enterica* serovar Typhimurium [20,21]. Notably, the bacterial QS system can crosstalk with mammalian genes through the perception of the AI-3/Epi/NE signal. Sperandio et al. found that the exogenous AI-3 and Epi restored the virulence phenotypes of the *luxS* mutant, but adrenergic antagonists can block this signal [22]. In addition to AIs, DSF and indole are two kinds of unusual QS signal molecules in *Salmonella*. Unlike other signaling molecules, the DSF and indole negatively regulate the virulence of *Salmonella*. DSF interacts with *Salmonella*’s master transcriptional regulator HilD, preventing the activation of *hilA* and inhibiting *Salmonella* invasion [23,24]. Indole, as an important microbiota metabolite, can attenuate bacterial motility, invasiveness, and virulence factors by reducing SPI-1 expression [25]; however, *Salmonella* cannot produce indole by itself.

In summary, although *Salmonella* cannot biosynthesize several QS signal molecules, the QS system plays a critical role in regulating the survival and virulence of *Salmonella*. For instance, the AI-2 produced by *Escherichia coli* can boost the oxidative stress response genes in *Salmonella*, increasing *Salmonella* pathogenicity [26]. This is consistent with the function of QS signaling molecules within *Salmonella*. Consequently, the QS signaling molecules function as a chemical signaling molecule that can affect *Salmonella* virulence on both intraspecific and interspecific levels.

## 3. Regulatory Mechanisms between QS and Virulence in *Salmonella*

The regulation of *Salmonella* virulence by QS involves a variety of processes. QS contributes to bacterial virulence by regulating the expression of genes involved in BF, flagella, T3SS, bacterial adhesion and invasion, and resistance to oxidative killing in macrophages (Figure 1). The role of various genes in QS regulation of *Salmonella* is listed in Table 1. The research on the regulation of QS on the virulence of *Salmonella* is helpful for attenuation *Salmonella* infections.

### 3.1. QS Affects BF-Mediated Virulence in Salmonella

Biofilm is a conglomeration of individual microbial cells which attach to a surface within a self-produced matrix of extracellular polymeric substances (EPSs) [43]. BF not only increases the virulence and persistent infection ability of *Salmonella*, but also enhances the development of antibiotic resistance, which plays an important role in *Salmonella* survival in adverse environments [44,45]. Some studies reported that *Salmonella* SdiA can utilize AI-1 molecules produced by other bacteria, which induce the formation of biofilm and affect its pathogenicity [46,47]. These data are supported by the fact that exogenously added AI-1-mediated QS molecules C4-HSL and C6-HSL, at a level of 1 µM, enhances BF as well as EPS secretion of *S*. Typhimurium [48]. In addition, BF can be triggered by AI-1-mediated QS molecules, C12-HSL, after 36 h under anaerobic conditions in *Salmonella enterica* Enteritidis [49]. This is consistent with the discovery that presence of C12-HSL enhances the expression of *glgC*, *fliF*, *lpfA*, and *fimF* genes which are implicated in the production of BF in *S*. Enteritidis [30,31]. The AI-2-mediated QS can also contribute to the formation of *Salmonella* biofilm through the expression of *luxS*. AI-2 disrupt BF in *S*. Typhimurium by reducing the expression of *luxS* [50]. The Epi-mediated QS not only affects the growth of *Salmonella*, but also impacts the BF of *Salmonella* under stress conditions in poultry breeding (especially in *Salmonella*-infected chickens) [51]. In the AI-3 QS system, it has been shown that BF is reduced when *qseB* are mutated, whereas *qseC* mutants were deficient in BF [52]. Thus, different QS signaling molecules promote the formation of biofilm in *Salmonella*, which facilitates its sustained colonization and exerts its pathogenic capacity.

### 3.2. QS Affects the Flagellar-Mediated Motility That Contributes to Virulence in Salmonella

Flagella, the important rotational propulsive organelles of *Salmonella*, play an important role in the adhesion and invasion of host cells, which contribute to *Salmonella* infection. The reduced expression of *sdiA* can decrease flagella formation (motility) and fimbria formation through affecting the expression of virulence factors controlled by QS, such as *pefI*/*srgC* operon, *srgE,* and *sirA* [53,54,55]. Additionally, it has been reported that the AI-2-mediated QS system affects *Salmonell*a flagellum formation which contributes to its virulence [35]. The expression of *luxS* and *fliC* decreased which influences the expression of flagella. It causes a reduction in *Salmonella* motility and affects the pathogenicity of *Salmonell*a. Furthermore, it has also been shown that overexpression of LsrR, a repressor which regulates and controls various genes in response to AI-2-P, in *Salmonella* substantially reduces the expression levels of *fliC*, *fliD*, as well as *flgL*, which have a critical function in flagellar formation and motility [5,22,35]. In addition, LsrK, the AI-2 kinase, plays an essential contribution to the regulation of flagellar photosynthesis and motility processes in *Salmonella* [35]. In the AI-3, Epi, and NE-mediated QS, various signaling molecules act as a global regulator of virulence by activating the expression of genes involved in flagellar biosynthesis and bacterial motility [56]. In indole-mediated QS, the signaling molecules of indole can inhibit bacterial flagella biosynthesis by reducing the expression of *flhC*, a major regulator of *Salmonella* flagellar biogenesis, which leads to decreased *Salmonella* motility [25]. In conclusion, different signaling molecules, mediated QS systems, decrease or increase the motility of *Salmonella* by affecting its flagella, which contributes to its virulence.

### 3.3. QS Regulates the Gene Expression of T3SSs Affecting Virulence in Salmonella

The T3SS system is encoded by SPI (*Salmonella* pathogenicity island) and constitutes the main vehicle for *Salmonella* to invade intestinal epithelial cells [57]. It plays a crucial role in its pathogenesis and virulence genes expression. SPI-1 is necessary for *Salmonella* intestinal invasion, while SPI-2 is necessary for *Salmonella* to proliferate in host cells [58,59]. SdiA, an AI-1 signaling molecule-mediated QS receptor, can regulate the expression of *Salmonella* virulence genes, such as *pefI*/*srgC* operon, *srgE,* and *sirA*, which in turn affect T3SS-dependent virulence, such as bacterial adhesion and invasiveness [60]. The addition of C12-HSL, an AI-1 signaling molecule, can increase the expression of *hilA*, *invA,* and *invF* of *Salmonella* SPI-1 [30]. In the AI-2 QS system, the AI-2 transporter complex, LsrR repressor, LsrABCD, and LsrK signaling kinase, constitute the *Salmonella* regulatory network [61]. LsrR negatively regulates SPI-1. Exogenous overexpression of the LsrR transcription factor reduces the expression of SPI-1. The levels of SPI-1 effector SipA, SipC, and SipD are also reduced, which is consistent with the regulatory effect of LsrR on SPI-1 [37]. In addition, deletion of LuxS reduces SPI-1 transcription and attenuates *Salmonella* invasion [35]. Furthermore, in the AI-3 QS system, the role of QseC is global as it regulates the gene expression of T3SSs in vitro and in vivo during mouse infection, thus affecting the virulence of bacteria [27]. On the other hand, indole signaling molecules inhibited SPI-1 expression in a concentration-dependent manner [25]. To summarize, the QS regulator of *Salmonella* plays an important role in regulating T3SS in different ways, which further controls the virulence of *Salmonella* against the host.

### 3.4. QS Regulates the Adhesion and Invasion of Host Cells Affecting Virulence in Salmonella

The invasion of host cells by *Salmonella* is considered to be the main feature of its pathogenesis. *Salmonella* can proliferate and survive once they enter host cells, thus triggering the onset of infection [62,63]. The AI-1-mediated QS receptor SdiA in *Salmonella*, which can bind to AHLs from other bacteria, regulates *rck*, which is implicated in microorganism adhesion and invasion of epithelial cells as well as resistance to the host complement [32]. SdiA also can regulate the genes located on the virulence plasmid and in the chromosome of *Salmonella,* and affects the bacterial invasion of host cells [29]. Moreover, it is reported that LsrR is a transcription factor that binds to AI-2 and can mediate the capacity of *Salmonella* to invade host cells [64]. Meanwhile, AI-2-mediated QS plays an important role in the invasion of *Salmonella* adhesion which contributes to *Salmonella* virulence. Studies show that the absence of AI-2 results in the diminished invasiveness of *Salmonella* against epithelial cells, which in turn attenuates its virulence in mice [36]. Deletion of the *luxS* (AI-2) synthetic gene weakened expression of *Salmonella* SPI-1 virulence genes, which are necessary for *Salmonella* to invade intestinal epithelial cells [36]. In addition, AI-3, Epi, and NE-mediated QS systems contribute to adhesion and invasion of *Salmonella* [65]. In the AI-3 QS system, when *qseC* is mutated, it can downregulate the transcription of virulence factors which reduced the ability of *Salmonella* to adhere and invade [52]. c2-HAD, a type of DSF, causes the loss of HilD activity by preventing the interaction of HilD with its target DNA in *S.* Typhimurium [23,24]. HilD forms a feed-forward loop with HilC and RtsA to induce HilA, which activates the expression of the SPI-1 to control invasion of epithelial cells [66]. Overall, *Salmonella* QS signaling molecules or their receptors regulate the adhesion and invasion of *Salmonella* to host cells, which contributes to the virulence of *Salmonella*.

### 3.5. QS Resists Oxidative Killing within Macrophages Affecting Virulence in Salmonella

Macrophages, as the main cells of *Salmonella* survival, have an important role in the process of host systemic infection caused by *Salmonella.* Therefore, one of the most important host defense systems that *Salmonella* must overcome is the process of invading bacteria-inducing oxidative bursts to produce bactericidal reactive oxygen species (ROS) [39]. It was proved that overexpression of LsrR can impede the evasion of oxidative killing in macrophages to make *Salmonella* virulence diminished [9]. Overexpression of *lsrR* leads to hypersensitivity to NADPH-dependent oxidative stress which inhibits the survival of *Salmonella* in macrophages [67]. Under *lsrR* overexpression conditions, the expression of oxidative stress-response genes such as *sodA*, *sodCI*, and *sodCII* decreased, resulting in *Salmonella* being highly sensitive to ROS, which is not conducive to the proliferation of *Salmonella* in macrophages [38,39]. It was revealed that epinephrine provides a clue to the proliferation of *S*. Typhimurium in macrophages [27]. Environmental cues for upcoming macrophage-derived peroxidation stress can be seen in the diminished survival of *S*. Typhimurium in macrophages to survive in the presence of adrenaline. In addition, NE and catecholamines support *Salmonella* survival in macrophages by mediating the expression of SPI-2 [27]. Thus, *Salmonella* senses its surrounding environment through QS signaling molecules, which help *Salmonella* to colonize and exert virulence beyond host defenses.

## 4. A New Strategy for Using the QS System to Regulate Bacterial Virulence

**Figure 2:**
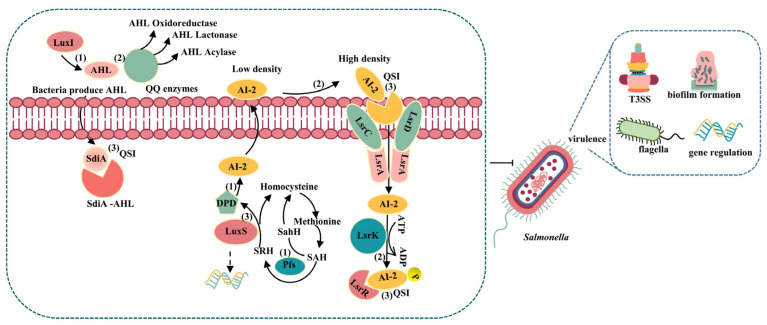
Strategies for QS interception. Strategies of QS inhibiting agents are marked with numbers on the diagram: (1) inhibit AIs synthesis; (2) inactivate AIs and degrade the enzyme of AIs; and (3) interfere with the AIs receptors.

Inhibition of QS systems not only leads to intensive reduction and regulation of severe infection but also reduces *Salmonella* infections and development of antibiotic resistance in humans. QS inhibition strategies are called “quorum quenching” (QQ) [19,68]. The interference of the QS process can be divided into the following three aspects (Figure 2), which are important tools to mitigate *Salmonella* infections and antibiotic resistance [69,70,71].

### 4.1. Inhibition of the Synthesis of Signal Molecules to Inhibit Virulence in Salmonella

The signaling molecules of QS perform an essential function in regulating physiological activities of bacteria [5,72]. *Salmonella* utilizes signaling molecules such as AI-2 as a communication tool to transmit interspecies information and express physiological characteristics [73]. The synthesis of QS signaling molecules requires the participation of various enzymes. For example, LuxS can catalyze the cleavage of the thioether bond degraded from SAH to SRH under the action of 50-methylthioadenosine/S-adenosylhomocysteine nucleosidase (Pfs), thereby generating 4,5-dihydroxyl-2,3-pentanedione (DPD), the precursor molecule of AI-2 [74]. Thereby, the synthesis of signal molecules can be inhibited by reducing the activity of related proteins or enzymes. Therefore, this may be a more direct way to interrupt the QS system and inhibit virulence factors such as biofilm by inhibiting the biosynthesis of QS signaling molecules.

### 4.2. Inactivation or Enzymatic Degradation of Signal Molecules to Inhibit Virulence in Salmonella

Signaling molecules can be degraded or inactivated by QQ enzymes [68,75,76]. Most QQ enzymes are involved in AHL inactivation or degradation, these include AHL lactonase/paraoxonase (lactone hydrolysis), AHL acyltransferase (amide hydrolysis), and AHL oxidase/reductase (oxidative reduction). AHL-lactonase has been identified to effectively inhibit bacterial BF and attenuate virulence factors [19]. While LuxS is a key enzyme that is directly involved in the production of AI-2, its inhibition will reduce the amount of AI-2. Furthermore, it has been reported that AI-2 is phosphorylated by LsrK and ATP, and because enzyme LsrK can phosphorylate DPD, this reaction phosphorylates AI-2 outside the cell and cannot pass through the cell membrane [77,78].

### 4.3. Inhibition of the Binding of Signal Molecules to Receptor Proteins to Inhibit Virulence in Salmonella

Inactivation or competition of receptors for QS signaling molecules can disrupt the QS system. Furanones, QS inhibitors, which can compete with native AI for binding and subsequently block AHL receptors, can significantly reduce bacterial virulence factor production and BF [19,79]. The synthesis of structural analogs with alkyl chains and aromatic rings from natural or chemically synthesized QSI (quorum sensing inhibitors) halogenated furanones can inhibit the formation of biofilm [80,81]. AI-2 and DPD analogs can compete with natural signaling molecules for binding to receptor proteins [82,83]. The binding of AI-2 and DPD to receptor proteins can be disrupted by inactivating LuxS [84,85]. Berberine inhibit certain QS behaviors by binding to LasR receptors and preventing the binding of proteins to DNA, thus exerting anti-infective effects [86].

## 5. Conclusions and Future Perspectives

The QS system not only plays an important role in *Salmonella* survival, but also controls *Salmonella* virulence [53]. Currently, anti-virulence therapy through the agents interfering with QS in *Salmonella* provides a strategy to prevent *Salmonella* infection and the development of antibiotic resistance [87,88,89]. Unlike antibiotics that kill bacteria to reduce bacterial infection, interfering with QS does not kill or inhibit the growth of bacteria, but rather resists *Salmonella* infection by interfering with the expression of virulence factors of bacteria [90]. Therefore, interference with QS in *Salmonella* has the advantage of lower survival pressure on bacteria, which can reduce bacterial antibiotic resistance. The development of QS inhibitors has become a novel strategy for the treatment of *Salmonella* infection and provides a new aspect for the virulence effect of *Salmonella*. QSIs such as furanone and its derivatives can be used to inhibit the virulence associated with *Salmonella*, which can reduce *Salmonella* infection. In addition, the synthetic QSI, N-phenyl-4-phenylamino-thioxomenthyl amino-benzenesulfonamide, can also inhibit *Salmonella* virulence [12,31]. Due to *Salmonella* being an intestinal pathogen, there are still some issues that deserve to be discussed. (1) What is the effect of QS interferer on the beneficial activity of host-associated microbiota during infection? (2) It is necessary to further understand whether QS directly drives pathogen virulence, or whether pathogens only use these events to indirectly create a favorable environment. (3) How fast does the resistance of *Salmonella* to QS interferers evolve? These issues will provide favorable conditions for the development of novel strategies to overcome, combat, or control *Salmonella* infection. In summary, considering the impact of *Salmonella* QS on bacterial virulence, it is helpful to control *Salmonella* infection and development of antibiotic resistance in the next generation of treatment to provide new insights.

## Figures and Tables

**Table 1:** Role of various genes in QS regulation of *Salmonella*.

Signals	Pathway	Role in Virulence Regulation	Reference
AHL	SdiA	Modulates expression of T3SS, increases biofilm formation and adhesion to epithelial cells, and regulates flagellin motility	[27,28,29]
	*glgC*/*fliF*/*lpfA*/*fimF*	Increases biofilm formation and modulates flagellin motility	[30,31]
	Rck region	Increases motility, adhesion, and invasion	[32]
	T3SS (*hilA*/*invA*/*invF*)	Enhances the expression of SPI-1	[30]
AI-2	LuxS	Regulates flagella, biofilm formation, and enhances the expression of SPI-1	[33,34]
	*lsrR*	Decreases expression of SPI-1 and flagella genes and regulates the invasion of *Salmonella* into epithelial cells	[35,36]
	*fliC/fliD/flgL*	Increases motility	[22,35]
	T3SS (*sipA*/*sipC*/*sipD*)	Enhances the expression of SPI-1 and regulates the invasion of *Salmonella* into epithelial cells	[37]
	*sodA/sodCI/sodCII*	Stress survival (oxidative stress)	[38,39]
AI-3/Epi/NE	QseBC	Regulates biofilm formation, flagella regulon, and increases adhesion	[40,41]
DSF	HilD	Inhibits invasion	[24]
Indole	*flhC*	Inhibits flagella biosynthesis	[42]
	SPI-1(*invF*/*hilA*)	Inhibits invasion	

## Data Availability

Not applicable.
